# Strontium isotopes reveal a globally unique assemblage of Early Miocene baleen whales

**DOI:** 10.1080/03036758.2023.2278732

**Published:** 2024-03-13

**Authors:** Felix G. Marx, Ambre Coste, Marcus D. Richards, J. Michael Palin, R. Ewan Fordyce

**Affiliations:** a Museum of New Zealand Te Papa Tongarewa, Wellington, New Zealand; b Department of Geology, University of Otago, Dunedin, New Zealand

**Keywords:** Mysticeti, Cetacea, Zealandia, dark age, dating, Aquitanian

## Abstract

The earliest Miocene (Aquitanian, 23–20 Ma) remains a critically under-sampled ‘dark age’ in cetacean evolution. This is especially true of baleen whales (mysticetes), Aquitanian specimens of which remain almost entirely unknown. Across the globe, the nature of the cetacean fossil record radically shifts at the Oligocene-Miocene boundary, with mysticetes and some archaic odontocete lineages suddenly disappearing despite the availability of cetacean-bearing rock units. New Zealand is the only place worldwide where this change is not readily apparent, with baleen whales apparently persisting into the earliest Miocene. Whether this is a genuine pattern has so far remained obscured by a lack of biostratigraphic resolution associated with the Oligo-Miocene boundary. Here, we report 23 new strontium (^87^Sr/^86^Sr) dates from *Lentipecten* shells associated with 16 mysticete and seven odontocete specimens, respectively. Of these, eight fall within the Early Miocene and seven – including five mysticetes – specifically within the Aquitanian. Our findings confirm the unique nature and global importance of the cetacean fossil record from New Zealand, and provide a foundation for investigations into the causes and effects of the Early Miocene cetacean ‘dark age’.

## Introduction

Baleen whales have a relatively rich fossil record stretching back to the late Eocene (Fordyce and Marx 2018; Muizon et al. 2019). Their evolutionary history can broadly be categorised into a Palaeogene phase, characterised by archaic forms with disparate feeding ecologies and a high degree of apparent endemism; and a Neogene phase, dominated by toothless cosmopolitan filter feeders (Marx et al. 2019; Hernández Cisneros and Velez-Juarbe 2020). The transition between these two phases remains a profound mystery, with baleen whales suddenly disappearing from the global fossil record at the Oligocene-Miocene boundary and remaining absent throughout the subsequent Aquitanian and, regionally, parts of the Burdigalian (Bianucci et al. 2018; Marx et al. 2019).

The absence of Early Miocene mysticetes cannot be explained by a simple lack of preservation, as contemporary deposits bearing odontocete fossils remain widespread across eastern and western North America (Barnes 1977; Gottfried et al. 1994; Crowley et al. 1999; Goedert et al. 2007; Whitmore and Kaltenbach 2008), western South America (Di Celma et al. 2018), Europe (Pilleri 1986a, 1986b; Bianucci and Landini 2002; Grigorescu and Kazár 2006), and Australia (Fitzgerald 2004; pers. comm.). Potential biotic or physical drivers also remain obscure but may relate to the selective extinction of archaic toothed species near major landmasses, with toothless forms (chaeomysticetes) persisting in offshore areas with a limited fossil record (Marx et al. 2019).

Here, we use strontium isotope dating to show that New Zealand rocks preserve a rich assemblage of Aquitanian mysticetes, the first of its kind anywhere in the world. We then discuss our findings in the globally unique geographical context of the sunken continent of Zealandia, and highlight implications for mysticete evolution in Australasia and beyond.

## Material and methods

### Specimen selection

To select specimens for analysis we surveyed the extensive vertebrate collections at the University of Otago, amassed by R.E.F. and colleagues over the past 40 years (Fordyce et al. 2015). We focused on baleen whale fossils to address the question of the Aquitanian ‘dark age’, but included several odontocetes to test the broader potential of New Zealand for Early Miocene discoveries. Two mysticetes from other collections were also included owing to their potential earliest Miocene ages: ZMT 67 from Canterbury Museum and WMA 2023/92 from Waitaki Museum. Specimens were chosen based on provenance and their association with well-preserved autochthonous pectinid shells (*Lentipecten* or its Miocene homeomorph sensu Beu et al. 2012), whose stable calcite shells are especially suitable for Sr dating (Nelson et al. 2004). In addition, we included a mysticete fossil that lacks associated pectinids but was stratigraphically correlated with a previously Sr-dated planktic foraminiferal sample (New Zealand Fossil Record File Database (www.fred.org.nz) fossil record number J39/f215 of Morgans et al. 1999; H. Morgans pers. comm.).

We focused on specimens from the Otekaike Limestone and the overlying Mount Harris Formation, which are well-exposed throughout the southern Canterbury Basin of the eastern South Island of New Zealand and straddle the Oligocene-Miocene boundary (Field and Browne 1986). The contact between these formations varies regionally and is not always clearly delineated. At the northwest face of Haughs/ Hakataramea Lime Quarry in South Canterbury, where many of the fossils analysed herein were found, massive shelly limestone grades up into a glauconitic and muddy limestone, changing from buff to brown in outcrop (Fig. S1) (Ksepka et al. 2023). This lithological change either suggests an unconformity or a condensed gradational boundary, as indicated by the dense shell beds beneath and at the contact. Based on our stratigraphic observations and dating results, we follow Tanaka and Fordyce (2014) in provisionally treating this as the contact between the Otekaike Limestone and Mount Harris Formation (Table 1).

**Table 1.** Strontium isotope ratios and inferred dates from Lentipecten shells associated with various Late Oligocene-Early Miocene cetacean fossil from New Zealand. ^1^Sr data derived from planktic foraminifera, from sample J39/f215 of Morgans et al. (1999). ^2^Specimen OU 22744 previously referred to cf. Waharoa (Boessenecker and Fordyce 2017b).

Description	Formation	Sample	87Sr/86Sr	2SE	Age (Ma)
Early Miocene, Burdigalian					
Chaeomysticeti indet.	Mt Harris Formation	WMA 2023/92	0.708569	0.000027	18.30 ± 0.40
Early Miocene, Aquitanian					
Chaeomysticeti indet.^1^	Mt Harris Formation	OU 22705	0.708373	0.000009	21.10 ± 0.15
Chaeomysticeti indet.	Mt Harris Formation	OU 22918	0.708294	0.000028	22.33 ± 0.53
Odontoceti indet.	Mt Harris Formation	OU 22773	0.708286	0.000025	22.45 ± 0.50
Chaeomysticeti indet.	Mt Harris Formation	OU 22733	0.708271	0.000024	22.73 ± 0.48
*Papahu taitapu*	Kaipuke Siltstone	OU 22066	0.708271	0.000027	22.73 ± 0.53
Chaeomysticeti indet.	Mt Harris Formation	ZMT 67	0.708270	0.000029	22.73 ± 0.58
Chaeomysticeti indet.	Otekaike Limestone	OU 22888	0.708256	0.000029	22.98 ± 0.58
late Oligocene, Chattian					
Odontoceti indet.	Otekaike Limestone	OU 22710	0.708251	0.000023	23.08 ± 0.48
Chaeomysticeti indet.^2^	Otekaike Limestone	OU 22744	0.708250	0.000027	23.08 ± 0.53
Odontoceti indet.	Otekaike Limestone	OU 22553	0.708249	0.000021	23.10 ± 0.45
Odontoceti indet.	Otekaike Limestone	OU 22709	0.708248	0.000027	23.13 ± 0.52
Chaeomysticeti indet.	Otekaike Limestone	OU 22381	0.708245	0.000019	23.15 ± 0.40
Odontoceti indet.	Otekaike Limestone	OU 21798	0.708233	0.000025	23.40 ± 0.50
Chaeomysticeti indet.	Otekaike Limestone	OU 22775	0.708223	0.000027	23.55 ± 0.55
Odontoceti indet.	Otekaike Limestone	OU 22540	0.708210	0.000012	23.80 ± 0.30
Chaeomysticeti indet.	Otekaike Limestone	OU 22404	0.708200	0.000015	23.98 ± 0.33
*Tohoraata raekohao*	Otekaike Limestone	OU 22178	0.708187	0.000014	24.20 ± 0.30
*Tokarahia kauaeroa*	Otekaike Limestone	OU 22235	0.708175	0.000015	24.43 ± 0.32
*Tokarahia* cf. *lophocephalus*	Otekaike Limestone	OU 22081	0.708166	0.000021	24.60 ± 0.45
Eomysticetidae indet.	Otekaike Limestone	OU 22783	0.708166	0.000020	24.60 ± 0.45
Chaeomysticeti indet.	Kokoamu Greensand	OU 22224	0.708137	0.000032	25.15 ± 0.65
*Waharoa ruwhenua*	Otekaike Limestone	OU 22044	0.708129	0.000016	25.30 ± 0.35

In addition to specimens from the Otekaike and Mount Harris formations, we analysed a mysticete from the Kokoamu Greensand, which underlies the Otekaike Limestone in the Canterbury Basin (Fordyce 2002); and one odontocete from Early Miocene Kaipuke Siltstone of the remote north-western coastline of the Tasman District, South Island (Aguirre-Fernández and Fordyce 2014).

We relied on ear bone anatomy to refer undescribed mysticete material to either Eomysticetidae, widely regarded as the basalmost chaeomysticete family (Boessenecker and Fordyce 2017a); or to a group of poorly resolved but seemingly more crownward lineages, here summarised as Chaeomysticeti indet (Tsai 2023). Anatomical characters considered included (i) the presence/ absence of fusion of the accessory ossicle on the periotic (fused in more crownward chaeomysticetes); (ii) the presence/ absence of a fused compound posterior process of the tympanoperiotic (fused in more crownward chaeomysticetes); (iii) the width of the anterior process of the periotic in ventral view (transversely compressed in eomysticetids); (iv) and the degree of medial rotation of the tympanic bulla (rotated in more crownward chaeomysticetes).

### Trace element analysis by LA-ICP-MS

Three to six *Lentipecten* shell fragments were selected per whale or dolphin fossil, mounted on glass slides, and encased in West System 105 epoxy resin blocks (briquettes) containing 10–13 fragments each. The briquettes were then ground down to the level of the shell fragments and polished.

To confirm the suitability of our samples for dating, we first tested for potential diagenesis via trace element analysis. Optically opaque or iron-stained areas in bivalve carbonate often reflect such post-depositional alteration (Bosio et al. 2020) and were specifically targeted early in this work to establish selection criteria for subsequent Sr isotope measurements. We chose conservative thresholds of Mn < 20 ppm and Sr > 700 ppm (Cummins et al. 2014).

Trace elements were measured using laser ablation inductively coupled plasma mass spectrometry (LA-ICP-MS) at the Otago Community Trust Centre for Trace Element Analysis at the University of Otago. We used a Resonetics (now Australian Scientific Instruments) RESOlution M-50-LR laser ablation system incorporating a Coherent CompexPro 102 193 nm ArF excimer laser and Laurin Technic two-volume sample cell. The laser was operated at a constant on-sample fluence of 2.5 J/cm^2^. Ablated material was carried by He gas (300 ml/min) from the two-volume sample cell, mixed with Ar (ca. 1000 ml/min) and N_2_ (3.5 ml/min), and input into an Agilent 7700cs quadrupole ICP-MS.

The quadrupole ICP-MS was tuned at the beginning of each day to achieve maximum sensitivity and stability with minimum oxide and doubly charged ion production. Data for 21 mass/charge ratios were collected in time-resolved mode with one point per ratio. Integration times were 10 ms for 22(44Ca++), 23Na+, 24Mg+, 27Al+, 29Si+, 31P+, 39K+, 43Ca+, 44Ca+, 55Mn+, 57Fe+, 59Co+, 65Cu+, 66Zn+, 75As+, 88Sr+, 202Hg+, 208Pb+, 220(background), 232Th+, and 248ThO + for a total scan time of 252 ms.

Background (laser off) data were acquired for 20 s followed by laser ablation for 40 s with a 7 Hz repetition rate, spot diameter of 75, and 10 µm/s tracking speed. This produced ablation tracks that were 75 µm wide × 400 µm long and yielded 150 mass scans. Three tracks were ablated in the optically clearest regions of 4–6 separate shell fragments for 12–18 total analyses for each sample.

Measurements for each sample were bracketed by analyses of NIST 610 glass and MACS3 carbonate standards. Raw mass peak count rates were background subtracted, corrected for mass bias drift, and converted to concentrations (in parts per million) by reference to the NIST 610 glass standard using an Excel spreadsheet offline. After triggering, a few seconds is needed for a steady signal to be reached so these initial data were excluded from the calculations. Trace-element concentrations were obtained by normalising count rates for each element to those for Ca in the sample and standard using known Ca and trace element concentrations in NIST 610 (Jochum et al. 2011) and assuming samples were pure CaCO_3_.

### Strontium isotope analysis by LA-MC-ICP-MS

Strontium isotopes were measured via laser ablation inductively coupled plasma multi-collector mass spectrometry (LA-MC-ICP-MS) at the Otago Community Trust Centre for Trace Element Analysis at the University of Otago. Laser operating conditions were as described above except that no N_2_ was added to the gas carrying ablated material into the Nu Plasma-HR MC-ICP-MS.

The MC-ICP-MS was tuned at the beginning of each day to optimise ion beam peak shape and sensitivity. Ion beams were measured simultaneously with an array of Faraday collectors with 10^11^ ohm resistors at mass/charge ratios of 89, 88, 87, 86.5, 86, 85.5, 85, 84.5, 84, 83, 82, and 81 for 89Y+, 88Sr+, 87Sr+, 173Yb++, 86Sr+, 171Yb++, 85Rb+, 169Tm++, 84Sr+, 83Kr+, 82Kr+, and 162Dy++. Ion beam intensities were recorded in volts (V) every 0.2 s.

Laser ablation was conducted with a 10 Hz repetition rate and spot diameter of 50 μm. Tracks on standards were ablated for 40 s at 10 µm/s (400 µm) yielding 200 measurements for each ion beam. Tracks on samples were ablated for 100 s at 5 µm/s (500 µm) yielding 500 measurements for each ion beam. Sample tracks were located to avoid areas of physical shell damage or possible alteration on the basis of optical characteristics and previous trace element analysis. Each track was pre-ablated at rapid speed to remove possible surface contamination followed by 40 s with the laser off to clear the sample cell and measure on-peak gas backgrounds for each ion beam.

Ion beam intensity data were processed off-line using an in-house Excel-based spreadsheet to generate ^87^Sr/^86^Sr ratios for each laser ablation track. The on-peak gas background levels were subtracted from all ion beam intensities to correct for both amplifier baselines and isobaric interferences from small amounts of Kr in the Ar. Additional isobaric interferences from doubly charged REE, Ca ± Ar dimers and Rb were corrected in sequence as described by Scanlan et al. (2018) although they were negligible for all the carbonates.

After the above isobaric interference corrections, the measured ^86^Sr/^88^Sr ratio was normalised to 0.1194 and the exponential mass fractionation law was used to correct instrumental mass fractionation. Once corrected, the data were normalised to the marine carbonate *Tridacna* clam (0.709176; Neymark et al. 2014), which is consistent with a value for NIST 987 of 0.710248 (McArthur et al. 2020).

The performance and accuracy of the above procedures have been verified by repeat analyses of international and in-house reference materials with known Sr isotope compositions. These include USGS basalt glasses BCR-2G, BHVO-2G, and BIR-1G, pyroxene JGG, and in-house anorthoclase KAN (Bonnington et al. 2023).

Uncertainties in the final ^87^Sr/^86^Sr ratios are reported at the 2σ level to account for sample-specific internal analytical error and the external error of simultaneously analysed reference materials. In addition, we report the mean squared weighted deviation (MSWD), which is a measure of scatter relative to the analytical errors of individual data points (Wendt and Carl 1991). An MSWD of 1 is ideal, with values >1.5 and <0.5 representing underestimates and overestimates of analytical error, respectively. Strontium isotope ratios were translated into numerical ages using the LOWESS 6C calibration curve of McArthur et al. (2020).

## Results and discussion

### Zealandia as a window into life offshore

Error-weighted means suggest Early Miocene strontium dates for eight of our fossils, with seven – including five mysticetes – dating to the Aquitanian (Table 1, S1). These results are globally significant, as they make New Zealand the only place worldwide with an unquestionable record of earliest Miocene baleen whales. Elsewhere, fossils from this time are either absent or likely just postdate the Aquitanian as, for example, in Argentina and South Australia (Pledge 2010; Raffi et al. 2020; Cuitiño et al. 2023).

The local persistence of mysticetes is likely explained by the unusual geographical setting of the ‘sunken continent’ of Zealandia (Strogen et al. 2023). By the late Oligocene, subsidence had reduced the latter to an isolated archipelago frequented by a variety of modern-looking chaeomysticetes, which comprised >80% of the local baleen whale assemblage (Marx et al. 2019). By contrast, the continental margins of Australia, North America and eastern Asia are dominated by archaic toothed lineages like aetiocetids, coronodonids and mammalodontids (Hernández Cisneros and Velez-Juarbe 2020; Boessenecker et al. 2023).

By the Oligocene-Miocene boundary any trace of these toothed forms disappears, leaving Aquitanian nearshore sediments worldwide devoid of mysticete fossils (Marx et al. 2019). Yet baleen whales as a whole clearly survived and hence presumably persisted offshore. Their absence from the fossil record can likely be explained by the fact that relevant deposits, formed far from the nearest landmass, mostly remain underwater.

Zealandia is an exception to this general pattern. Unlike transient oceanic island archipelagos, whose fossil record is quickly obliterated by erosion, the continental nature of submerged New Zealand allowed it to be tectonically rejuvenated by plate boundary collision and upraised in large scale from the Oligocene to present day (Graham 2015). This resulted in the exposure of a suite of marine sediments that provide a window into life offshore and, thus, a mysticete fossil assemblage generally not recorded elsewhere. From this offshore habitat, baleen whales eventually recolonised nearshore waters, leading to their re-appearance in the global fossil record during late Early and Middle Miocene.

### Disappearance of eomysticetids

Eomysticetids are among the basalmost chaeomysticetes and notably contribute to global Oligocene baleen whale assemblages (Boessenecker and Fordyce 2017a; Hernández Cisneros and Nava-Sanchez 2022). Intriguingly, they are the only mysticete lineage that appears equally well-represented in late Oligocene onshore and offshore settings, with 15% of records from south-eastern Australia and 23% of those from New Zealand belonging to this group (Marx et al. 2019). Given this cosmopolitan distribution, the question arises how – if at all – eomysticetids were affected by the Oligocene-Miocene nearshore turnover event that led to the extinction of other stem baleen whales.

The youngest undoubted eomysticetid in our dataset, *Tohoraata raekohao*, dates to 24.20 ± 0.30 Ma and thus predates both OU 22744 and the Oligocene-Miocene boundary by more than one million years. Eomysticetids from elsewhere are of similar age or older, with the youngest – *Eomysticetus whitmorei* from the Chandler Bridge Formation of South Carolina (USA) – dating to at least 23.5 Ma and, plausibly, ≥24.5 Ma based on oyster-derived Sr dates (Weems et al. 2016). However, there are some possible exceptions, including (i) a fragmentary specimen from New Zealand (OU 22744) previously dated to the earliest Miocene and referred to cf. *Waharoa* (Boessenecker and Fordyce 2017b); (ii) some purportedly Burdigalian material from the Vaqueros Formation exposed at Laguna Canyon in California, USA (Rivin 2010); and (iii) isolated ear bones from Belgrade Quarry in North Carolina possibly as young as 22.8 Ma (Boessenecker 2022).

**Figure 1.**
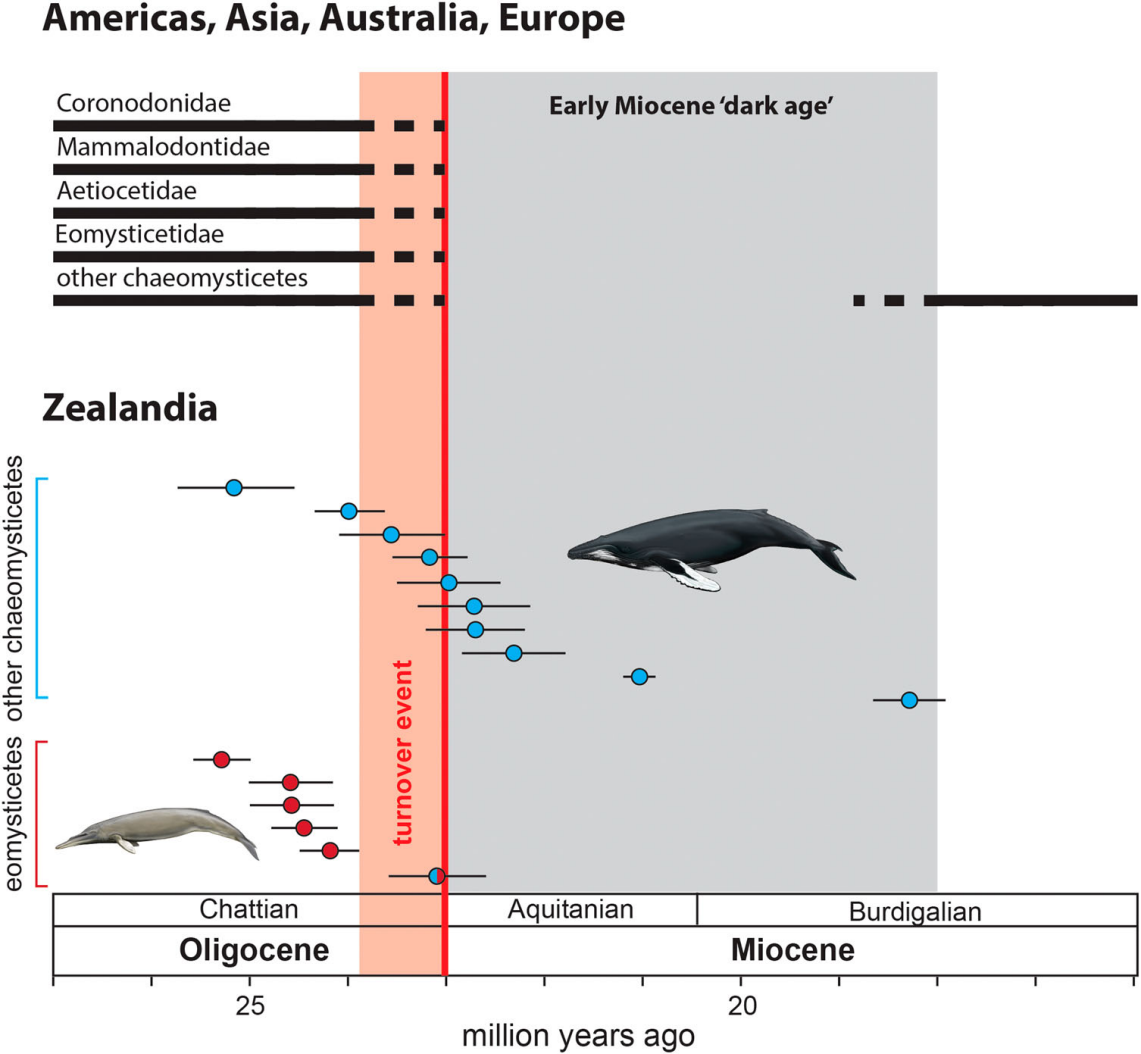
Occurrence of baleen whale fossils in the Americas, Asia, Australia and Europe (top) and in the rock record of Zealandia (bottom). The ages and associated errors of the New Zealand data reflect our novel ^87^Sr/^86^Sr dates. Note the global disappearance of all mysticetes during the Early Miocene gap, with stem lineages (coronodonids, aetiocetids, mammalodontids and eomysticetids) seemingly going extinct and other chaeomysticetes only reappearing during the Burdigalian. In New Zealand, eomysticetids (red circles) are also restricted to the Oligocene and may have disappeared more than one million years prior to the Oligocene-Miocene boundary. By contrast, more crownward mysticetes (blue circles) persist into the Early Miocene without obvious interruption. The specimen showing both colours is OU 22744, previously referred to the eomysticetid cf. *Waharoa* (Boessenecker and Fordyce 2017b) but here identified as a potentially more crownward chaeomysticete (see text). Artwork by Carl Buell.

In their original description of cf. *Waharoa*, Boessenecker and Fordyce (2017b) cautioned that OU 22744 ‘does not clearly display any obvious eomysticetid synapomorphies’ and thus mainly based their assignment on phenetic similarities of the robust atlas. Since their study, preparation of further undescribed material (e.g. OU 22888) has shown that a robust atlas bone also occurs in several Oligocene-Miocene boundary fossils that are clearly outside Eomysticetidae and share features with crown baleen whales (e.g. accessory ossicle fused to the anterior process of the periotic; fused posterior processes of the tympanoperiotic). We thus suggest that the identification of OU22744 is inconclusive and that this specimen may not, in fact, represent an eomysticetid. Even if it did, our associated strontium date of 23.08 ± 0.53 Ma lies just within the Oligocene and is thus slightly older than previously suggested (Figure 1, Table 1).

Besides eomysticetids, the Vaqueros Formation at Laguna Canyon has yielded toothed mysticetes that elsewhere have only been recorded from the Oligocene (Rivin 2010). The proposed Miocene age of this assemblage is based on the occurrence of apparently Hemingfordian (20–16 Ma) rodents in the underlying Sespe Formation (Rivin 2010). However, the reliability of these fossils as biostratigraphic markers is hampered by the highly variable anatomy of rodent teeth (Heuck 2019). Marine invertebrates from the Vaqueros Formation at Laguna Canyon date to either the late Oligocene or earliest Miocene and, consequently, are also of limited stratigraphic use (Heuck 2019). Overall, there is thus no compelling evidence for a Miocene age of the Vaqueros Formation and the archaic cetacean assemblage within.

Finally, the isolated eomysticetid ear bones from Belgrade Quarry are derived from spoil piles and, thus, of uncertain stratigraphic provenance. Their likeliest source – the Belgrade Formation – seemingly spans the Oligocene-Miocene boundary, with maximum and minimum ages of 25.95 and 22.82 Ma, respectively (Boessenecker 2022). The Belgrade Formation has further yielded a mixed marine mammal fossil assemblage that hints at either a genuinely transitional Oligocene-Miocene fauna, or time averaging of distinct Oligocene and Miocene source horizons (Boessenecker 2022). Given their uncertain provenance and the fact that the Belgrade Formation largely aligns with the Oligocene, we conservatively interpret the eomysticetids from Belgrade Quarry as predating the Oligocene/Miocene boundary.

While not entirely conclusive, available age data suggest that eomysticetids disappeared alongside other stem mysticetes at the end of the Oligocene. This observation potentially challenges the idea that the Early Miocene ‘dark age’ represents a nearshore phenomenon, given that eomysticetids occur in offshore settings as well. It is possible, however, that the loss of its nearshore habitat still affected the family as a whole, especially in light of its relatively low abundance compared to both toothed and other more crownward chaeomysticetes (Marx et al. 2019).

## Conclusions

We have shown that New Zealand rocks preserve numerous Early Miocene baleen whale fossils – the first such assemblage worldwide. Our results are consistent with previous suggestions of an Oligocene-Miocene turnover event that manifested in nearshore waters and led to the extinction of toothed mysticetes and, probably, eomysticetids. Further work is required to establish the scope and ramifications of this event, e.g. in terms of its impact on other groups like archaic odontocetes. In addition, more precise dating of fossil mysticete assemblages elsewhere should reveal if the event was sudden or gradual, and whether it coincided with – or preceded – the Oligocene-Miocene boundary.

## Supplementary Material

Table S1

Supplementary Material

## Data Availability

The full results of the strontium analysis are provided as Supplementary Material.
