# Supplementary material for: Strontium isotopes reveal a globally unique assemblage of Early Miocene baleen whales
Source: J R Soc N Z. 2024 Mar 13;54(5):711–21. doi: 10.1080/03036758.2023.2278732 (PMC11459727; doi:10.1080/03036758.2023.2278732)
Supplement: Supplementary Material [file TNZR_A_2278732_SM0322.docx]

**Supplementary Material**

Marx FG, Coste A, Richards M, Palin JM, Fordyce RE 2023. Strontium isotopes reveal a globally unique assemblage of Early Miocene baleen whales. Journal of the Royal Society of New Zealand, in review


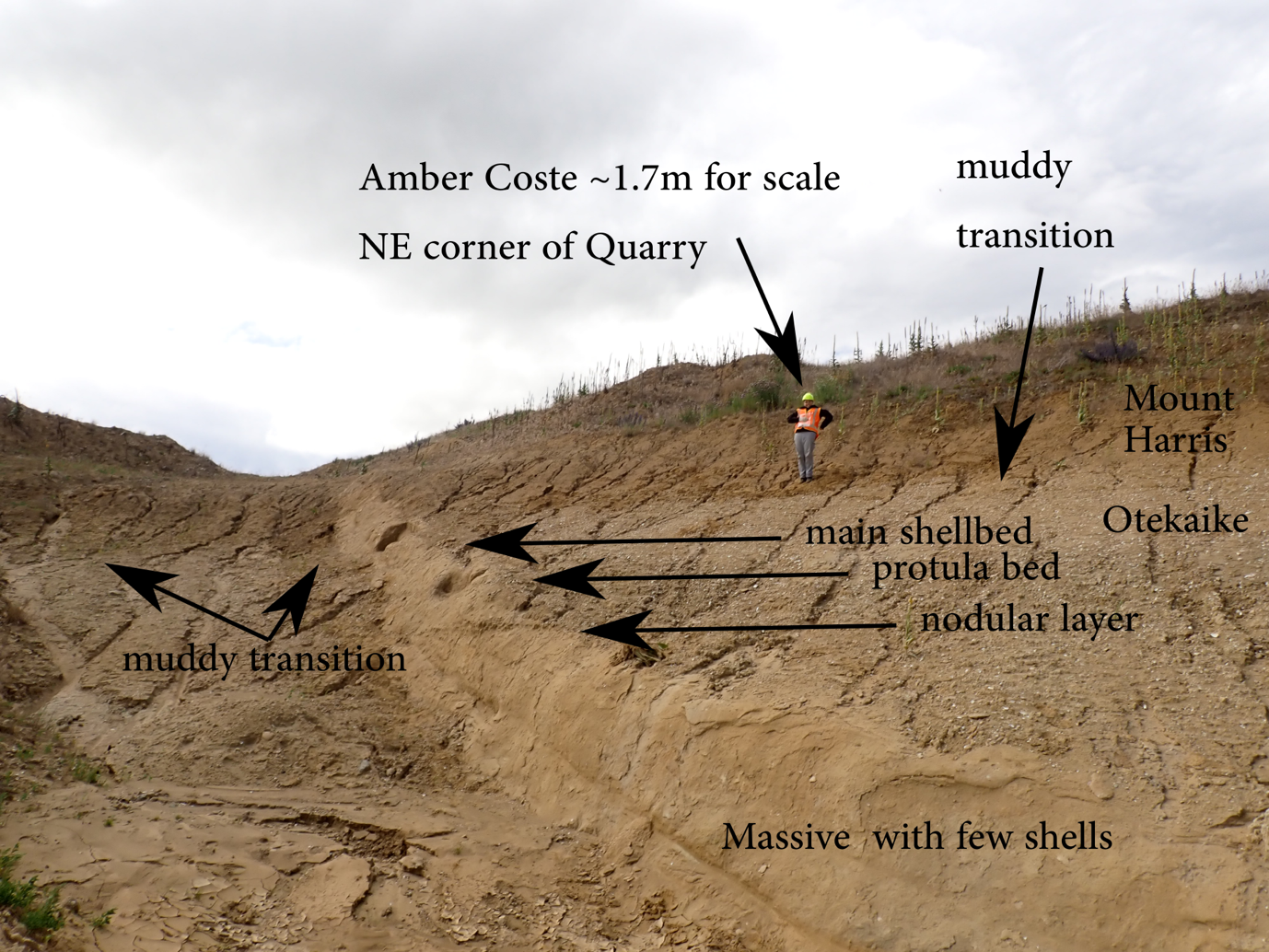


**Supplementary Figure S1.** Stratigraphy exposed at the top of Haughs’ Quarry, Hakataramea Valley, South Canterbury. Author A. Coste for scale. Note the change from Otekaike Limestone to overlying brown, muddy limestone of Mount Harris Formation at the level of A. Coste’s feet.
